# The membrane-spanning domain of gp41 plays a critical role in intracellular trafficking of the HIV envelope protein

**DOI:** 10.1186/1742-4690-7-95

**Published:** 2010-11-13

**Authors:** Kosuke Miyauchi, A Rachael Curran, Yufei Long, Naoyuki Kondo, Aikichi Iwamoto, Donald M Engelman, Zene Matsuda

**Affiliations:** 1 Laboratory of Virology and Pathogenesis, AIDS Research Center, National Institute of Infectious Diseases, 1-23-1 Toyama, Shinjuku, Tokyo, Japan; 2 Department of Molecular Biophysics and Biochemistry, Yale University, Box 208114, New Haven, CT 06520-8114, USA; 3 China-Japan Joint Laboratory of Structural Virology and Immunology, Institute of Biophysics, Chinese Academy of Sciences, 15 Datun Road, Beijing, 100101 PR China; 4 Division of Infectious Diseases, Advanced Clinical Research Center, University of Tokyo, 4-6-1 Shirokanedai, Minato-ku, Tokyo, Japan; 5 Research Center for Asian Infectious Diseases, Institute of Medical Science, University of Tokyo, 4-6-1 Shirokanedai, Minato-ku, Tokyo, Japan; 6 Current Address: Department of Pediatrics, Emory University School of Medicine, 2015 uppergate Dr. Atlanta, GA 30322, USA

## Abstract

**Background:**

The sequences of membrane-spanning domains (MSDs) on the gp41 subunit are highly conserved among many isolates of HIV-1. The GXXXG motif, a potential helix-helix interaction motif, and an arginine residue (rare in hydrophobic MSDs) are especially well conserved. These two conserved elements are expected to locate on the opposite sides of the MSD, if the MSD takes a α-helical secondary structure. A scanning alanine-insertion mutagenesis was performed to elucidate the structure-function relationship of gp41 MSD.

**Results:**

A circular dichroism analysis of a synthetic gp41 MSD peptide determined that the secondary structure of the gp41 MSD was α-helical. We then performed a scanning alanine-insertion mutagenesis of the entire gp41 MSD, progressively shifting the relative positions of MSD segments around the helix axis. Altering the position of Gly694, the last residue of the GXXXG motif, relative to Arg696 (the number indicates the position of the amino acid residues in HXB2 Env) around the axis resulted in defective fusion. These mutants showed impaired processing of the gp160 precursor into gp120 and gp41. Furthermore, these Env mutants manifested inefficient intracellular transport in the endoplasmic reticulum and Golgi regions. Indeed, a transplantation of the gp41 MSD portion into the transmembrane domain of another membrane protein, Tac, altered its intracellular distribution. Our data suggest that the intact MSD α-helix is critical in the intracellular trafficking of HIV-1 Env.

**Conclusions:**

The relative position between the highly conserved GXXXG motif and an arginine residue around the gp41 MSD α-helix is critical for intracellular trafficking of HIV-1 Env. The gp41 MSD region not only modulates membrane fusion but also controls biosynthesis of HIV-1 Env.

## Background

HIV-1, the retrovirus responsible for the current worldwide AIDS pandemic, is an enveloped virus. The envelope protein (Env) of HIV-1 is essential for determining host range and for inducing the membrane fusion that allows the virus to enter the host cell. The former and latter functions are mediated by the SU (gp120) and the TM (gp41) subunits of the envelope protein, respectively [1-3]. The SU and TM are generated from a precursor (gp160) by cellular proteases that recognize a basic amino acid sequence between gp120 and gp41 [4-6]. This proteolytic processing is essential to generate fusion-competent HIV-1 Env and is believed to take place in an early Golgi region [7,8].

HIV-1 Env is anchored across lipid bilayers via its highly conserved membrane-spanning domain (MSD) [9]. Although the possibility of a transient alteration of the membrane topology exists [10,11], HIV-1 Env is widely believed to be a type I membrane protein with a single α-helical MSD in the steady state [12]. Two different models exist within the single MSD model of HIV-1 Env. In an initial model, the MSD is supposed to be 23 amino acid residues long, ranging from Lys683 to Val704 in the HXB2 sequence, and has a highly conserved hydrophilic arginine residue in the midst of its hydrophobic amino acid sequence [13]. In an alternative model, MSD is shorter; and the arginine residue in the lipid bilayer is expected to interact with the polar head of the lipid molecule [14,15].

The primary structure of the MSD of HIV-1 Env also has a GXXXG motif, a motif often found at the helix-helix interface of transmembrane α-helices [16]; it exists upstream of the arginine residue. If an ordinary α-helix structure is assumed for the MSD, the GXXXG motif and arginine residue are positioned on opposite sides of the gp41 MSD α-helix.

In vitro studies of the gp41 MSD showed a high tolerance for mutations. For example, the above mentioned conserved arginine residue [17] and the GXXXG motif can accommodate point mutations [18]. Even several heterologous MSDs can replace the entire gp41 MSD without deteriorating effects [17,19]. These findings led to the notion that the specific amino acid sequence in the gp41 MSD has no significant biological role within the limits of the assays used. This is a curious notion since the sequence is quite conserved in nature, despite the virus being subject to very strong sequence diversification from errors in reverse transcription.

In fact, other studies have suggested that the specific sequence of the gp41 MSD plays a role in the function of gp41 [20,21]. We have shown that replacing the gp41 MSD with MSDs derived from glycophorin A or VSV-G, each containing the GXXXG motif, severely decreases the fusion activity of HIV-1 Env [18,22]. Furthermore, simultaneous substitution of all three glycine residues, within the GXXXG motif with leucine residues, also negatively affected the function of the HIV-1 Env [23]. Shang et al. recently showed the importance of the GXXXG region using a unique genetic approach [24]. These studies clearly suggested the presence of important information encoded in the sequence of MSD. However, the nature of the code is still not evident.

To further elucidate the structure-function relationship of the gp41 MSD, we analyzed a circular dichroism (CD) profile of the synthetic peptide corresponding to the MSD and obtained the profile expected for α-helical secondary structure. Next, we used the envelope gene of HXB2 [25] to create a series of alanine insertion mutants of the entire predicted MSD. We found that alteration of the relationship between Gly694 and Arg696 (the number indicates the position of the amino acid residues in HXB2 Env) around the axis of the MSD α-helix resulted in fusion incompetent Env. These mutant Envs also showed defects in proteolytic processing and intracellular transport in the endoplasmic reticulum (ER) and Golgi regions. We further showed that the intracellular transport of HIV-1 Env is regulated by the MSD region, through experiments that transplanted the gp41 MSD into another membrane protein, Tac. This transplantation led to an alteration of the intracellular distribution of Tac, similar to that of HIV-1 Env.

## Results

### Circular dichroism analysis of the synthetic MSD peptide in lipid shows α-helical secondary structure

**Figure 1:**
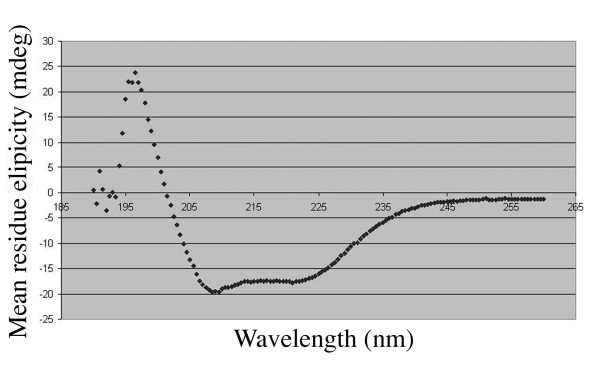
**The circular dichroism (CD) profile of the synthetic MSD peptide**. The synthetic peptide was dissolved in 15 mM DPC (n-dodecyl pyridinium chloride), 20 mM NaPi, 150 mM NaCl. The spectrum information was collected as described in the materials and methods section. The diagram shown is the average of eight spectra.

The primary structure of the gp41 MSD is highly conserved, and its secondary structure has been predicted to be an α-helix based on computational algorithms [26]. However, there are no physical data to support this expectation. We synthesized a peptide corresponding to a consensus HIV-1 clade B structure of the gp41 MSD and determined its CD spectrum in lipid bilayers. The CD profile, shown in Figure 1, has negative maxima near 208 nm and 222 nm, indicating the presence of an α-helical structure. Although the gp41 MSD of HIV-1 contains three glycine residues, thought to be helix-breaking residues in soluble proteins, the dominant structure indicated by our CD data was an α-helix. Many glycines are found in transmembrane helices. Addition of lysine residues at both ends was necessary to allow us to purify the extremely hydrophobic MSD peptide. We cannot completely exclude the possibility that these lysine residues at the termini, especially at the C-terminus, may stabilize the α-helical structure.

### Scanning alanine-insertion mutagenesis identified the region of gp41 MSD critical for membrane fusion

**Figure 2:**
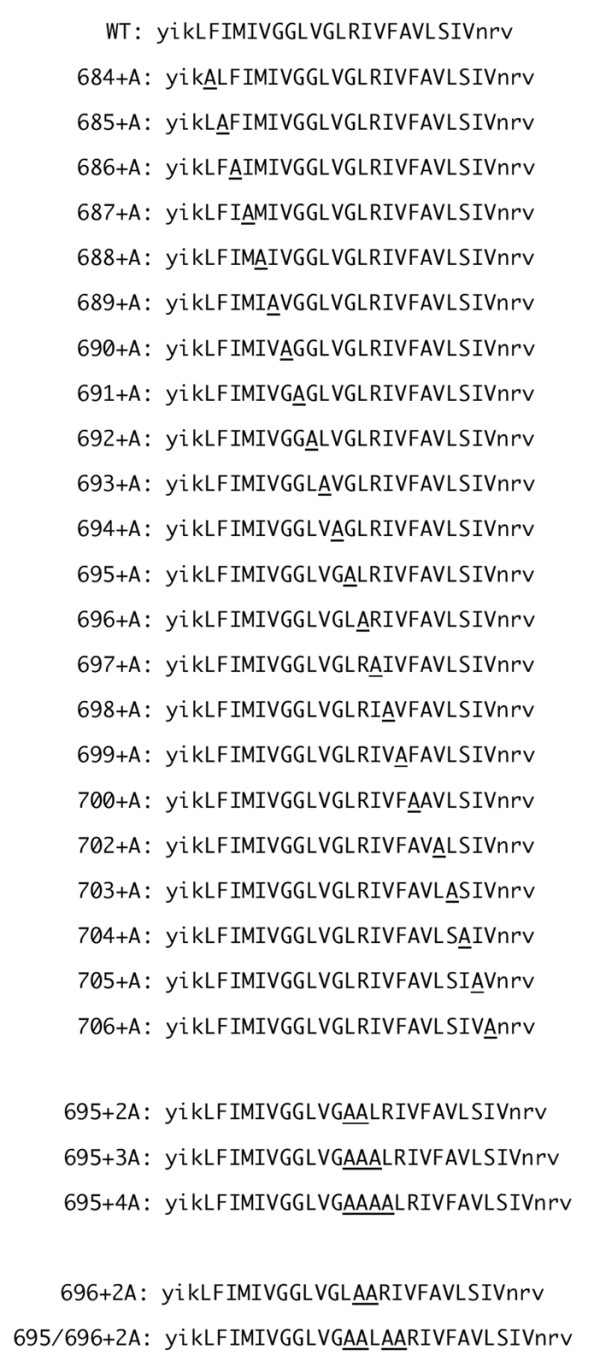
**Amino acid sequences of the MSD of the wild type (WT) and Ala-insertion mutants used in this study**. The predicted MSD portion is indicated in capital letters. The inserted alanine residue is underlined.

**Figure 3:**
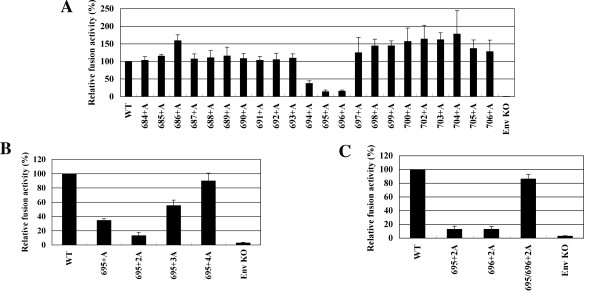
**The fusion activity of Ala-insertion mutants in the cell-cell fusion assay**. COS-7 cells transfected with the T7 RNA polymerase expression vector and the Env expression vector were co-cultured with 293CD4 cells transfected with a plasmid containing T7 promoter-driven renilla luciferase reporter. After a 24-hr co-culture, the renilla luciferase reporter activity was measured and normalized to the firefly activities as described previously [18]. The normalized renilla luciferase activities for (*A*) single Ala-inserted mutant of Env, (*B*) the mutant Env with multiple Ala insertion, (*C*) mutant Env with two alanine residues inserted at positions 695 and 696 are shown. Data are the average of three independent experiments. The error bar indicates a standard error.

To identify the region of the gp41 MSD α-helix critical for its function, we generated a set of alanine-insertion mutants covering the entire predicted MSD by using the HXB2 envelope gene. The alanine residue was chosen because it can be well accommodated in an α-helix [27,28]. Since previous data suggest the involvement of the gp41 MSD in membrane fusion [18,23,24,29], membrane fusion activity was determined for the mutants. The primary structures of these mutants are shown in Figure 2. Nomenclature is based on the positions of the inserted alanine residues in HIV-1 Env. Therefore, 684+A mutant indicates that the inserted alanine residue corresponds to the 684th residue of the envelope protein. The mutant envelope gene was cloned into the envelope expression vector, and the fusion activity of each mutant was determined by the T7 RNA polymerase transfer assay as described previously [18]. The result is shown in Figure 3A. Among the twenty-two mutants we generated, three showed a prominent decrease in the fusion activity. These three are 694+A, 695+A, and 696+A; their relative fusion activities when compared with the wild type (WT) were 37.5%, 14.0% and 15.5%, respectively. Mutants 695+A and 696+A showed more severe defects than 694+A. Thus the corresponding region from 694 to 696, the G^694^LR^696 ^region, was shown to be critical for fusion activity.

### The alteration of the phase of the GLR region in MSD was critical to membrane fusion

The insertion of an alanine residue affects both the length and the phase of the α-helix. However, we expected that the local phase might have a more important role than the length of the MSD because all the insertion mutants generated are expected to have the same length of the MSD. To verify this, we inserted one, two, three, and four alanine residues between residues 694 and 695 (Figure 2 bottom, 695+2A, 3A, and 4A) and examined the fusion activities of each resulting mutant. The result is shown in Figure 3B. The insertion of two residues caused a further decrease in the fusion activity compared to the single insertion (compare 695+A and 695+2A). However, the fusion activity slightly recovered with the insertion of three residues, and almost fully recovered by the insertion of four alanine residues. It seems that there is a correlation between the recovery of the phase of gp41 MSD α-helix and recovery of membrane fusion activity. The observed defect in fusion activity was not due to the increase in length but instead to the local shift of the gp41 MSD α-helix.

To further identify residues critical for determining the phase in the GLR region, we generated 696+2A and 695/696+2A (a combination of 695+2A and 696+2A, Figure 2 bottom) and then compared the fusion activity together with 695+2A. Both 695+2A and 696+2A showed severe defects in membrane fusion (Figure 3C). Interestingly, the combination of these two (695/696+2A) recovered fusion activity. The phase commonly altered in the fusion defective mutants, 695+2A and 696+2A, but corrected in the fusion competent 695/696+2A mutants was found to be between Gly694 and Arg696. Thus the relationship between Gly694 and Arg696 seems to be an important factor for the membrane fusion activity.

### Analysis of the protein profile of the fusion-defective mutants reveals impaired processing of gp160 into gp120 and gp41

**Figure 4:**
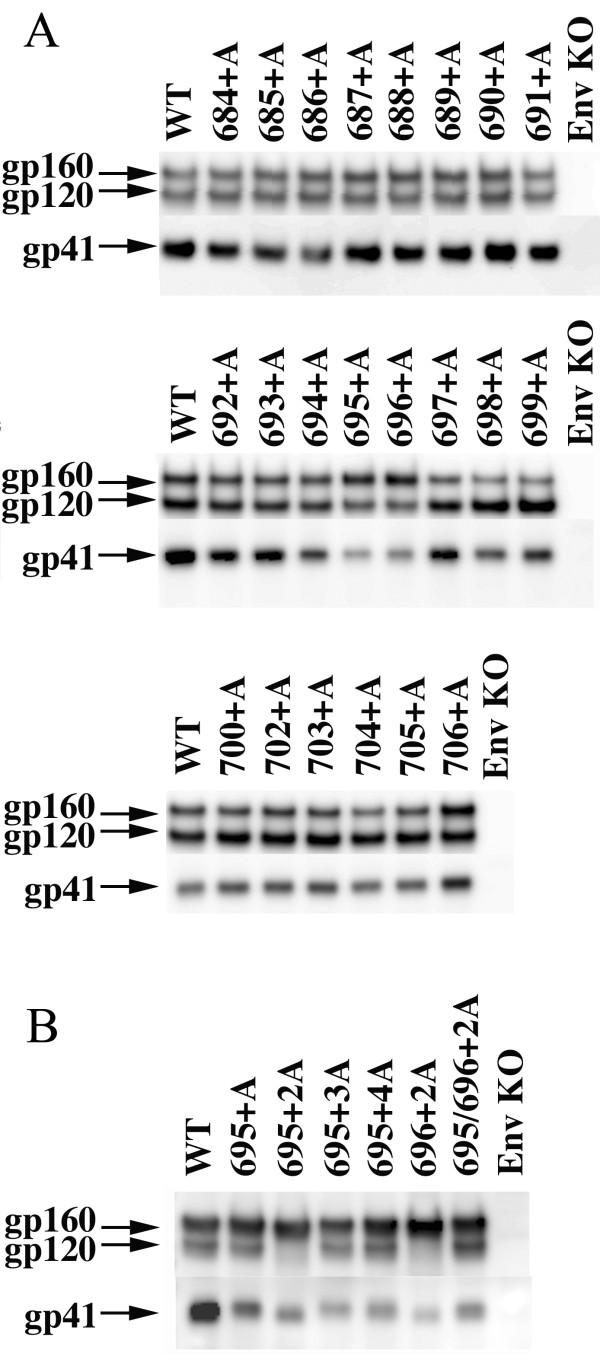
**The immunoblotting analysis of wild type (WT) and Ala-inserted mutant Env**. The envelope proteins expressed in COS-7 cells transfected with the Env expression vector were detected with anti-gp120 antibody (for gp160 and gp120) or with anti-gp41 antibody. The results of single- and multiple-Ala-insertion mutants are shown in (*A*) and (*B*), respectively.

We analyzed the protein profiles of these mutant Envs by immunoblotting, using anti-gp120 and anti-gp41 antibodies (Figure 4). All mutant Envs were expressed at comparable levels (Figure 4A); however, the fusion-defective mutants had impaired processing of gp160, namely more gp160 than gp120; and accordingly less gp41 (see 694+A, 695+A, and 696+A) was observed. This tendency was more prominent for 695+2A and 696+2A, each of which showed severe defects in fusion. A similar correlation between impaired processing of Env and defective membrane fusion was observed in the multiple alanine insertion mutants that showed defective fusion (Figure 3C and 4B). Because the generation of processed gp41 is a prerequisite for fusion competency, this protein profile for inefficient gp160 processing is consistent with the observed fusion defect. Our data showed that the alteration in the α-helical phase in the localized region within gp41 MSD affected processing of gp160 into gp120 and gp41. It was also shown that these mutants were fusion incompetent. A possibility is that the mutations induced allosteric structural changes of the cleavage site so that the mutant Env was no longer processed properly by Furin or Furin-like proteases. However, this idea was not supported by the observation that mutant gp160, purified from COS-7 cells, is cleaved into the gp120 and gp41 subunits by commercially available Furin *in vitro *(Additional file 1). We also analyzed the trimerization of Env mutants. The trimer of 695+2A Env was detected (Additional file 2) However, the presence of less drastic yet critical structural alterations by the mutation cannot be ruled out completely.

### Alanine insertion in the gp41 MSD can alter the intracellular distribution of Env

**Figure 5:**
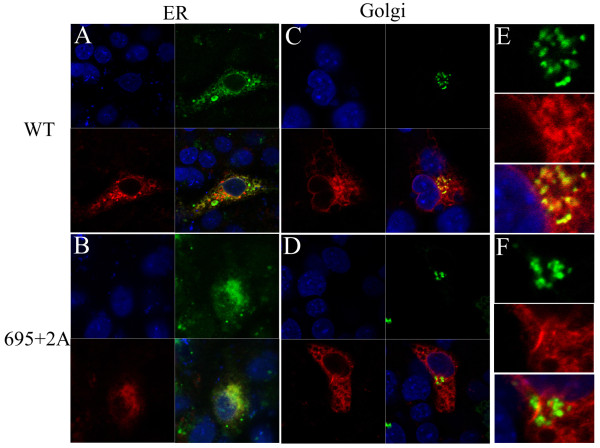
**The transport defect of alanine insertion mutant Env**. Endoplasmic reticulum (ER) (A and B) and Golgi (C to F) regions were visualized by fluorescence protein-conjugated ER or Golgi marker proteins (shown in green). FLAG tagged WT (A, C and E) and 695+2A Env (B, D and F) were stained by anti-FLAG antibody and Alexa Fluor (shown in red). The close-up of the Golgi area was shown in E and F. Nuclei of cells were stained with Hoechst 33258 (shown in blue).

**Figure 6:**
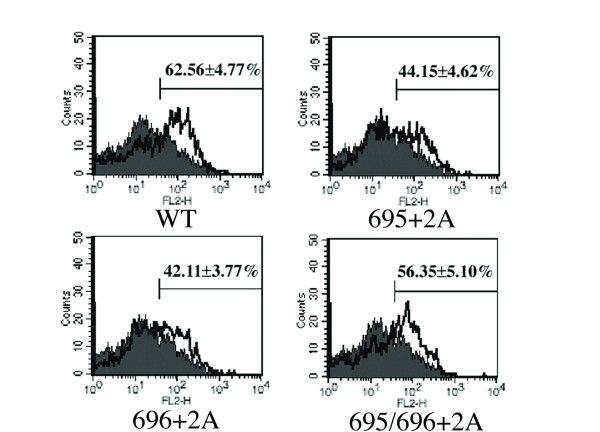
**Surface expression level of Env**. The cell surface expression level of envelope proteins for WT and Ala-insertion mutants on transfected COS-7 cells was determined by flow cytometry using anti-gp120 antibody.

Since processing of gp160 takes place in the Golgi [7,8], we hypothesized that the defect in the processing was derived from the defect in the intracellular trafficking of mutant Env in the endoplasmic reticulum and Golgi regions. To test this possibility, we examined the distribution of mutant Env in the cells. We attached a FLAG tag at the C-terminus of gp41, providing a linear epitope that can be recognized by monoclonal antibody, M2. An ttachment of the FLAG tag did not alter the defect in processing present in alanine insertion mutants (data not shown). The envelope proteins expressed in COS-7 cells were visualized by immunofluorecent assay using the anti-FLAG monoclonal antibody. We observed that fine, mesh-like fluorescent signals distributing within the transfected cells were more prominent for the mutant 695+2A than the WT (Figure 5). The intensity of fluorescence derived from Env at the Golgi area was notably weaker for 695+2A than for the WT. These data suggested that mutant Env was defective for transport from ER to Golgi. The level of Env expressed on the cell surface, analyzed by FACS, is consistent with this observation because it is lower for the mutant than for the WT (Figure 6).

**Figure 7:**
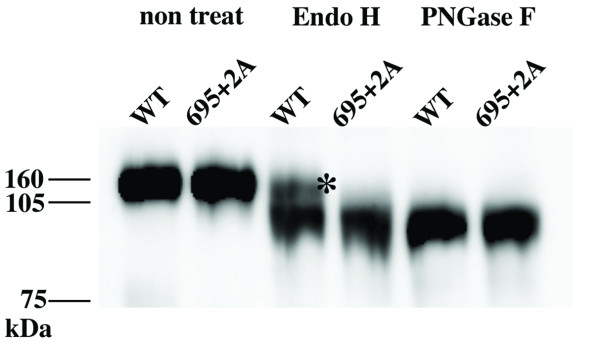
**The analysis of glycosylation of WT and mutant Env**. The FLAG-tagged Env purified from transfected COS-7 cells was treated with Endo H or PNGase F glycosidase. The treated protein was separated by SDS-PAGE and detected by immunoblotting analysis using anti-FLAG antibody. The asterisk shows the endo H-resistant fraction of Env.

To further verify the transport defect biochemically, we analyzed the pattern of modification of sugar moieties in the WT and mutant Env. The results are shown in Figure 7. When treated with endoglycosidase H (Endo H), the WT exhibited an Endo H-resistant fraction of gp160 whereas almost no Endo H-resistant gp160 was detected in the 695+2A mutant. This finding indicated that sugar moieties attached to the mutant envelope protein remained as high-mannose types. However, both the WT and mutant envelope proteins generated bands that migrated similarly after treatment with Peptide: N-Glycosidase F (PNGase F), which cleaves between the innermost GlcNAc, and asparagine residues, where sugar moieties are attached. These data further confirmed the defect of the mutant envelope protein in transport, probably in ER-Golgi regions.

### The transfer of the gp41 MSD into a foreign membrane protein alters the intracellular distribution of chimeric proteins

**Figure 8:**
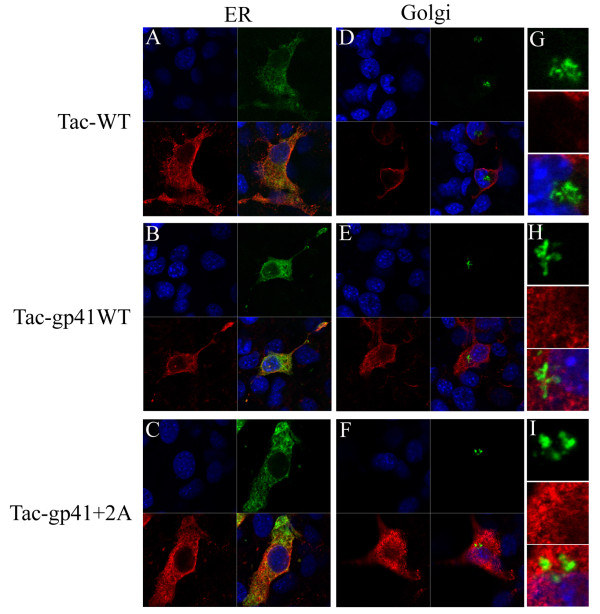
**Intracelluar distribution of Tac-gp41MSD chimera**. The influence of MSD in transport of Tac proteines. Endoplasmic reticulum (ER) (A to C) and Golgi (D to I) regions were visualized by fluorescence protein-conjugated ER or Golgi marker proteins (shown in green). Halo tagged Tac-WT (A, D and G), Tac-gp41WT (B, E and H) and Tac-gp41 695+2A Env (C, F and I) were stained by anti-Halo antibody, anti-rabbit Ig and Alexa Fluor (shown in red). The close-up of the Golgi area was shown in G to I. Nuclei of cells were stained with Hoechst 33258 (shown in blue).

We are interested in determining whether the MSD region alone is sufficient to induce the observed transport defect in the context of other membrane proteins. To test this possibility we have replaced the MSD of Tac, the α-chain of the Interleukin-2 receptor, with the MSD of the wild type (Tac-gp41WT) or 695+2A mutant (Tac-gp41+2A) of gp41, and determined the intracellular distribution of the engineered Tac proteins. We included the intact Tac as a reference (Tac-WT). The results are shown in Figure 8. The signals of intact Tac proteins distributed both in the cytoplasm and plasma membrane areas. They show a fine mesh like appearance in the cytoplasm and are well overlapped with the signals of the ER markers. The intact Tac proteins also showed prominent signals at the rim of the cells suggesting efficient transport to the plasma membrane (Figure 8A and 8D). There was no overlap of signals for intact Tac and Golgi markers (Figure 8G). When the MSD of intact Tac proteins was replaced with that of gp41 (wild type in Figure 8B and 8E; 695+2A in C and F), the signals corresponding to the plasma membrane areas became weaker than those of intact Tac (Figure 8, compare A with B and C; D versus E and F). The majority of the signals was observed in the cytoplasm, and the signals were co-localized with ER markers (Figure 8B and 8C). There are some signals of Tac-gp41 chimera in Golgi areas (Figure 8H and 8I). Different from the context of HIV-1 envelope proteins (Figure 5E and 5F), we did not detect a discernable difference in the distribution between the wild type gp41 MSD (Figure 8 Tac-gp41WT) and 695+2A gp41 MSD (Tac-gp41+2A) in the Golgi areas (Figure 8H and 8I). It appeared that the introduction of the gp41 MSD made chimeric Tac distribute in the cytoplasmic region, mainly ER regions, but the difference between the wild type gp41 and 695+2A mutant became less prominent in the context of Tac than in the context of the HIV-1 Env.

## Discussion

Although the gp41 MSD has three glycine residues, our CD analysis suggested the presence of the α-helical structure in gp41 MSD (Figure 1). This may not be a surprise, since glycines are abundant in transmembrane helices and glycines are viewed as helix breakers in soluble proteins. A recent molecular dynamics study also supports a helical conformation [30]. Furthermore, the replacement of all three glycine residues with alanine residues, highly α-helix-forming residues [27,28], did not affect the fusion activity of gp41 [18]. Thus gp41 MSD is presumably functional with an α-helical structure. These data, however, do not rule out the possibility of the reported transient alteration of the secondary structure of the gp41 MSD during membrane fusion [11].

Our scanning alanine insertion mutagenesis identified the topological relationship between Gly694 and Arg696 around the MSD α-helix as a critical determinant for the proper processing (Figure 4) and intracellular transport (Figure 5) of Env. Since the processing of gp160 is dependent on the proper transport of the proteins to the Golgi apparatus, it seemed that the observed defect in processing might be due to a transport defect. However, we cannot rule out the possibility of a potential allosteric structural alteration of the Env by mutation in the MSD as a cause for the inefficient processing. Indeed our recent data suggested that the mutations in the gp41 MSD exert allosteric conformational changes of the ectodomain of HIV-1 Env [22].

Mutation at the cleavage site of gp160 eliminates HIV-1 Env fusogenicity [7]. Thus, the defective membrane fusion of our alanine insertion mutants seemed to be derived from improper processing of gp160. However, there are other factors contributing to the defective fusion. Many studies have shown that mutations in the gp41 MSD affect membrane fusion efficiency [18,23,24,29]. In the context of 695+2A mutant, the substitution of hydrophilic arginine residue with non-polar residues (alanine or isoleucine) rescues the defective processing (Additional file 3); however, this could not resolve the defective fusion (Additional file 4). These data suggest that gp41 MSD has a role(s) in the membrane fusion process itself. To reveal the exact mechanism, further studies are required.

It has been reported that MSD length is crucial for the trafficking of membrane proteins [31]. In HIV-1 Env, length of the MSD alone does not seem to be a primary determinant for trafficking. However, our data show that critical information lies in the local structure of the transmembrane α-helix of gp41. It is possible that the alteration of structural features in the MSD region can be sensed by host factor(s) involved in the protein quality control system. This detection could be through the MSD region itself. In a yeast system, some proteins involved in the vesicular transport in ER-Golgi where target recognition was achieved via the MSD region have been reported [32,33]. Since the distribution of our Tac-gp41 chimera was heavily affected by the replacement of the MSD region alone (Figure 8), it may support such a hypothesis. Such a hypothetical factor may recognize wild type gp41 MSD via the GXXXG motif facing outward in relationship to the MSD bundle, if the gp41 MSDs interact with each other through arginine residues as suggested recently [30].

Notably, our alanine insertion mutation altered the relative positioning of the GXXXG motif and arginine residue within the gp41 MSD. Both are major interaction motifs between transmembrane α-helices [34,35]. Although recent electron cryomicroscopic data [36-38] did not provide a spatial arrangement of the gp41 MSD portions, it is possible that there are interactions between the gp41 MSDs during the biosynthesis of the HIV-1 Env. Our alanine insertion may disrupt the interaction among MSDs. This disturbance of interhelical interactions may result in altered intracellular transport. The failure to reproduce differences in intracellular distribution between the wild type and 695+2A MSD, in the context of Tac (Figure 8, B-I), may arise from the difference in the oligomeric status between HIV-1 Env (trimer) and Tac (monomer). Our data suggest that mutant Env still forms a trimer (Additional file 2).

Our data clearly demonstrate that the MSD of gp41 has important functions in the biosynthesis of HIV-1 Env, apart from the simple anchoring and modulation of fusion efficiency. The exact regulation mechanism of intracellular distribution of HIV-1 Env by the MSD portion is not known; however, it could be of great importance to determine whether there are any cellular factors that specifically recognize the MSD region of HIV-1 Env.

## Conclusions

We have shown that the secondary structure of the synthetic peptide of gp41 MSD is an α-helix. Based on this information, we performed a scanning alanine insertion mutagenesis which showed that alteration of the topological relationship between conserved GXXXG motif and the arginine residue resulted in non-functional Env. The mutant Env manifested a reduced fusion activity and impaired the processing of gp160 into gp120 and gp41. Furthermore, the intracellular transport of mutant Env was affected in the endoplasmic reticulum and Golgi areas. Our data suggested that the specific α-helical structural feature of gp41 MSD controls the biosynthesis of HIV-1 Env.

## Methods

### Synthesis of MSD peptides and its circular dichroism analysis

The sequence of the peptide used is KKWYIKIFIMIVGGLVGLRIVFAVLSIVNRKK, which corresponds to the consensus sequence of predicted MSD of clade B HIV-1. The sequence of the MSD of the clade B molecular clone, HXB2, used in this study differs by one amino acid from this sequence (indicated by the underline, HXB2 has L instead of I at this position). Two lysine residues were introduced at the N- and C-termini to make the peptide more hydrophilic. The CD spectra were measured at 25°C with Aviv Model 215 (Aviv biomedical Inc,, Lakewood, NJ) in 15 mM DPC (n-dodecyl pyridinium chloride), 20 mM NaPi, 150 mM NaCl. The concentration of the peptide was 10 μM. Eight spectra were averaged after subtracting for a DPC reference sample.

### Generation of the MSD mutants

QuikChange Site-Directed Mutagenesis kit (Stratagene, La Jolla, CA) generated the mutants used in this study. The plasmid, pGEM7zNB, which contains the 1.2-kb *Nhe*I-*Bam*HI fragment covering the *env *portion of HXB2RU3ΔN, was used as a template as described previously [18]. To facilitate the mutagenesis, silent restriction enzyme sites for *Hind*III, *Spe*I, and *Bsiw*I were generated near the MSD coding region. The complementary oligonucleotide pairs containing an inserted codon, GCC, for the alanine residue were cloned by using the *Hind*III, *Spe*I, and *Bsiw*I sites. Multiple Ala-insertion mutants were made based on the single-insertion mutants. The complementary oligonucleotide pairs used were: 695+A, GGAGGCTTGG TAGGTGCTTT AAGAATAGTT TTT/AAAAACTATT CTTAAAGCAC CTACCAAGCC TCC, 696+A, GGCTTGGTAGGTTTAGCTAGAATAGTTTT TGCT/AGCAAAAACTATTCTAGCTAAACCTACCAAGCC,695+2A, GAGGCTTGGTAGGTGCTGCCTTAAGAATAGTTTTTGC/GCAAAAACTATTCTTAAGGCAGCACCTACCAAGC CTC,695+3A, GTAGGAGGCTTGGTAGGTGCGGCCGCATTAAGAATAGTTTTTGCTGTACGTACAGCAAAAACTATTCTTAATGCGGCCGCACCTACCAAGCCTCCTAC, 695+4A, GGAGGCTTGGTAGGTGCGGCCGCAGCCTTAAGAATAGTTT TTGCTGTAC/GTACAGCAAAAACTATTCTTAAGGCTGCGGCCGCACCTACCAAGCCTCC,696+2A, GCTTGGTAGGTTTAGCTGCCAGAATAGTTTTTGCTG/CAGCAAAAACTATTCTGGCAGCTAAACCTACCAAGC,695/696+2A, GAGGCTTGGTAGGTGCTGCCTTAGCTGCCAGAATAGTTTTTGCTG/CAGCAAAAACTATTCTGGCAGCTAAGGCAG CACCTACCAAGCCTC. The *Nhe*I-*BamH*I fragment of pGEM7zNB containing the expected mutations was cloned back to pElucEnv [18] or pElucEnv-3FLAG Env (see below) expression vectors.

The synthetic codon-optimized gene corresponding to the Tac protein, α-chain of Interleukin-2 receptor, with the gp41 MSD was custom synthesized (GenScript, Piscataway, NJ). The derivatives of this construct, whose MSD portion was replaced with those of wild type or mutant gp41 or intact Tac, were generated by mutagenesis using PCR. These genes were cloned downstream of the CMV promoter to generate the Tac-derivative expression vectors.

### Addition of the 3 × FLAG tag at the C-terminus of the Env

A 3 × FLAG tag was added to the C-terminus of gp41 by inserting oligonucleotides corresponding to the 3 × FLAG tag sequence derived from the vector p3xFLAG-CMV™-7.1 (Sigma, St. Louis, MO). Following this insertion, the amino acid sequence after the C-terminus of gp41 reads as RSARDYKDHDGDYKDHDIDYKDDDDK. The expression vector of FLAG-tagged Env was called pElucEnv-3FLAG Env.

### Cells and antibodies

COS-7 cells, 293 cells, and 293-CD4 cells [18] were grown in Dulbecco's modified Eagle's medium (Sigma, St. Louis, MO) supplemented with 10% fetal bovine serum (HyClone Laboratories, Logan, UT) and penicillin-streptomycin (Invitrogen, Carlsbad, CA). Cells were kept under 5% CO_2 _in a humidified incubator. Anti-gp120 polyclonal antibody was obtained from Fitzgerald Industries International, Inc. (Concord, MA). The hybridoma 902 and Chessie 8 were obtained from Bruce Chesebro and George Lewis, respectively through the AIDS Research and Reference Reagent Program, Division of AIDS, National Institute of Allergy and Infectious Diseases, National Institutes of Health, USA [39-41]. Anti-FLAG M2 and BioM2 were purchased from Sigma (St Louis, MO).

### Cell-cell fusion assay

Cell-cell fusion assays, using T7 RNA polymerase (T7 RNA pol) transfer, were performed as described previously [18]. Briefly, 293-CD4 cells that constitutively express CD4 were transfected with pTM3hRL harboring the T7 promoter-driven renilla luciferase gene by FuGene 6 (Roche Applied Science, Mannheim, Germany), and were co-cultured with COS-7 cells that had been transfected with pCMMPT7iresGFP, a T7 RNA polymerase expression vector, and pElucEnv containing HIV-1 Env and firefly luciferase genes by FuGene 6. After 12 hours of co-culture, the renilla and firefly luciferase activities were measured using the Dual-Glo luciferase assay system (Promega, Madison, WI). The fusion activity, represented by renilla luciferase activity, was normalized by firefly luciferase activity to obtain transfection efficiency [18]. The polyclonal anti-halo antibody was obtained from Promega (Promega, Madison, WI).

### Immunoblotting analysis

5 × 10^4 ^COS-7 cells were transfected with pElucEnv by FuGene 6 in a 24-well culture plate. Forty-eight hours after transfection, the cells were lysed with radioimmunoprecipitation assay lysis buffer (0.05 M TrisCl, 0.15 M NaCl, 1% Triton X-100, 0.1% sodium dodecyl sulfate, and 1% sodium deoxycholate). Cell lysates were electrophoresed (5-20% Pantera Gel, DRC, Tokyo, Japan) and transferred to a polyvinylidene fluoride membrane (Pall, East Hills, NY). The blot was probed with anti-gp120 antibody (Fitzgerald, Concord, MA), with the monoclonal anti-gp41 antibody (Chessie 8), or with anti-FLAG M2 antibody. A biotinylated anti-species-specific immunoglobulin (GE Healthcare Bio-Sciences AB, Uppsala, Sweden) was used as the secondary antibody. The blot was further treated with a streptavidin-horseradish peroxidase conjugate (GE Healthcare Bio-Sciences AB) and Lumi-Light^plus ^(Roche, Indianapolis, IN). Images were obtained with LAS3000 (Fujifilm, Tokyo, Japan).

### Immunofluorescence assay

Immunofluorescence assays were used to determine the intracellular distribution of the envelope proteins. For this purpose, we generated a modified envelope expression vector called pElucEnvdeltaGFP; this is the derivative of the previously described pElucEnv [18] and it has the deletion of the EGFP portion. COS-7 cells transfected with pElucEnv WT or 695+2A in the delta GFP backbone vector and ER-DsRed2 or Golgi-YPF (Clontech) or pER-mAG1 (MBL, Nagoya, Japan) plasmid by FuGene 6 (Roche) were treated with PBS including 4% of PFA for 5 min at 48 hr posttransfection. Cells were permeabilized by PBS including, 0.05% of saponin and 0.2% of BSA, for 30 min and then stained with 20 μg/ml of bio-M2 (Sigma) antibody and 10 μg/ml of streptavidin conjugated Alexa fluor 488 or 555 (Invitrogen). In the case of Halo-tagged proteins, polyclonal anti-Halo antibodies were used as primary antibodies. The distributions of fluorescence in cells were visualized using a Zeiss LSM 510 meta confocal microscope.

### Flow cytometric analysis

Flow cytometric analysis was performed as described previously [18]. Briefly, COS-7 cells were transfected with pElucEnv by FuGene 6 on a six-well plate. Forty-eight hours aftertransfection, the cells were stained with anti-gp120 monoclonal antibody 902, biotinXX anti-mouse IgG (Invitrogen) and streptavidin-Alexa 555 in PBS including 10% FBS. Cells were fixed with 1% paraformaldehyde in PBS and analyzed by FACS Calibur (BD Biosciences).

### Glycosidase assay

COS-7 cells transfected with pElucEnv-3FLAG by FuGene 6 on the six-well plate were lysed with radioimmunoprecipitation assay lysis buffer including Complete protease inhibitor (Roche). Env-3FLAG was purified from cell lysates by immunoprecipitation using M2 agarose (Sigma) and eluted with 3XFLAG peptide (Sigma). Purified Env-3FLAG was treated with Endo H or PNGase F (Roche). For digestion by Endo H, Env-3FLAG was boiled and digested with 0.005 unit Endo H at 37°C for 12 hr in Endo H digestion buffer [50 mM phosphate buffer (pH 5.8), 50 mM NaCl, 0.1 M 2-mercaptoethanol (2-ME), 0.01% SDS]. Env-3FLAG was boiled in PBS including 0.1 M 2-ME and 0.1% SDS and digested by 1 unit PNGase F at 37°C for 12 hr in PNGase F digestion buffer (74 mM TrisCl, pH 8.0; 0.74% NP-40; 0.37 M 2-ME, 0.37% SDS). Env-3FLAG treated with glycosidase was resolved by SDS-polyacrylamide gel electrophoresis (10% polyacrylamide gel; DRC) and detected by immunoblotting analysis using anti-FLAG M2.

### In vitro furin cleavage of Env

Env-3FLAG with the 695+2A mutation was purified from COS-7 cell lysates by immunoprecipitation as described above and treated with 0.7 units of furin (Alexis, Lausen, Switzerland) at 30°C for 12 hr in furin-digestion buffer (100 mM Hepes, pH 7.5; 1 mM CaCl_2_; 0.5% Triton X-100]). Env-3FLAG, treated with furin, was detected by immunoblotting analysis using anti-FLAG M2 as described above.

### The cross linking analysis of Env

At 48 hr postransfection, 293T cells transfected with FLAG tagged WT or 695+2A Env expression vectors were treated with 1 mM DSS for 20 min at room temperature in PBS (pH 8.0). Cells were incubated with 20 mM Tris-Cl for 15 min at room temperature to stop the reaction and then were lysed in buffer A (10 mM HEPES, 1.5 mM MgCl2, 10 mM KCl, 0.5 mM DTT, 0.05% Igepal pH 7.9). Env proteins in cellular lysate were detected by immunoblotting analysis using anti-FLAG antibody (see above).

## List of abbreviations

MSD: membrane-spanning domain; CD: circular dichroism; ER: endoplasmic reticulum; WT: wild type.

## Competing interests

The authors declare that they have no competing interests.

## Authors' contributions

**KM, ARC, YL **and **NK **performed most of the experimental work. **KM, YL **and **NK **did the cell biological analyses of mutant Envs. ARC analysed the synthetic peptide for its biophysical properties. **AI **contributed to discussion. **DME **and **ZM **conceived the study and coordinated the experiments. All authors read and approved the final manuscript.

## Supplementary Material

Additional file 1 **Supplemental Figure 1- In vitro digestion of mutant Env with recombinant Furin**. The wild type (WT) and mutant (695+2A) Env were prepared from transfected COS-7 cells and subjected to digestion with recombinant Furin (rFurin) as described in the Methods section. Mock indicates the result for the cell lysates prepared from mock transfected cells. Click here for file

Additional file 2 **Suplemental Figure 2- Cross linking analysis of the 695+2A Env**. The trimerization of gp160 was examined by chemical cross linking. The cells transfected with Env expression vectors for wild type (WT) and mutant (695+2A) were treated with the chemical cross linker. The cell lysates were probed with the anti-FLAG antibody. The single asterisk and the double asterisk indicate the bands for trimer and monomer of mutant gp160, respectively. Marker: HiMark Pre-Stained High Molecular Weight Protein Standard (Invitrogen), Mock: mock transfection. Click here for file

Additional file 3 **Suplemental Figure 3A - Immunoblotting analysis of the Arg-substitution mutants in the context of 695+2A**. The degree of processing of gp160 was examined by immunoblotting the cell lysates prepared from COS-7 cells transfected with respective Env expression vectors. The Arg residue in the context of 695+2A was substituted with the indicated amino acid residue by the site directed mutagenesis (columns under 2A). One letter abbreviation for an amino acid residue is used. Mock: mock transfection, WT: wild type MSD. Click here for file

Additional file 4 **Suplemental Figure 3B - Fusion activities of Arg-substitution mutants in the context of 695+2A**. The fusion activities of the mutant shown in additional file 3A were examined by a syncytia formation assay in 293CD4 cells. Fusion activity of the WT and MSD mutants was expressed using a fusion index (fusion index = 2x + y, where x is the number of multinucleated cells [number of nuclei ≥ 5 in five visual fields] and y is the number of multinucleated cells [number of nuclei < 5 in five visual fields]) as described previously [18]. Click here for file
