# Supplementary figures and images for: The membrane-spanning domain of gp41 plays a critical role in intracellular trafficking of the HIV envelope protein
Source: Retrovirology. 2010 Nov 13;7:95. doi: 10.1186/1742-4690-7-95 (PMC2994783; doi:10.1186/1742-4690-7-95)

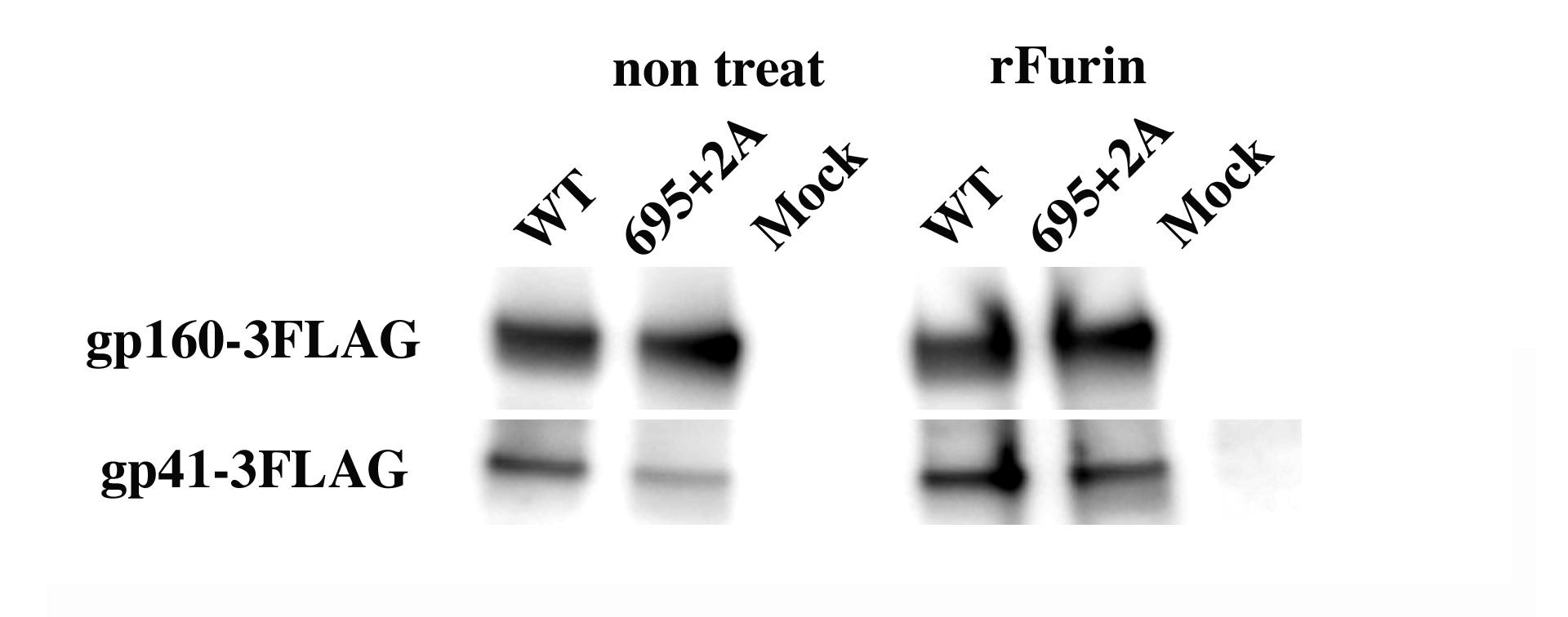

Supplement: Additional file 1 — Supplemental Figure 1- In vitro digestion of mutant Env with recombinant Furin. The wild type (WT) and mutant (695+2A) Env were prepared from transfected COS-7 cells and subjected to digestion with recombinant Furin (rFurin) as described in the Methods section. Mock indicates the result for the cell lysates prepared from mock transfected cells. [file 1742-4690-7-95-S1.TIFF]

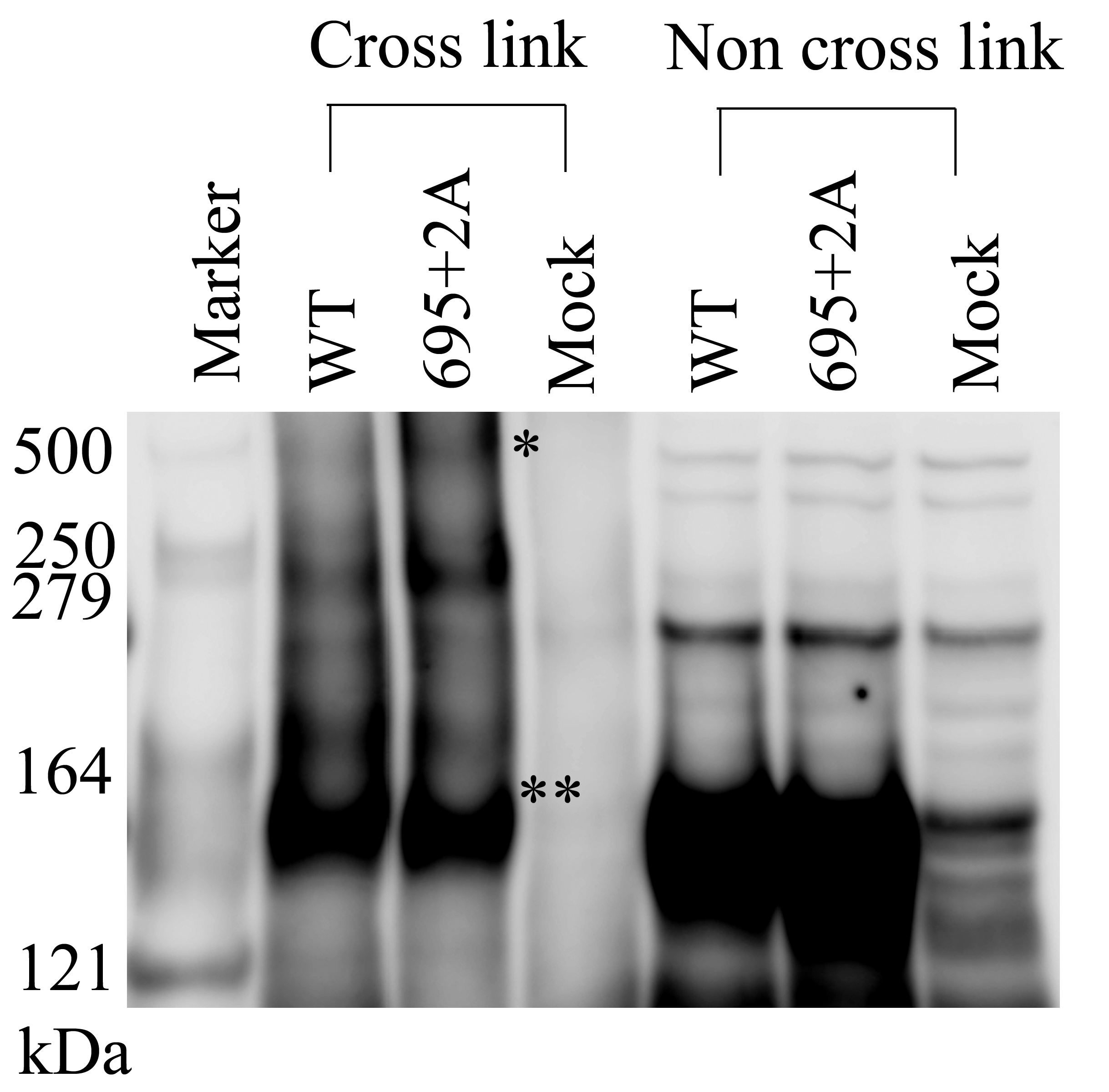

Supplement: Additional file 2 — Suplemental Figure 2- Cross linking analysis of the 695+2A Env. The trimerization of gp160 was examined by chemical cross linking. The cells transfected with Env expression vectors for wild type (WT) and mutant (695+2A) were treated with the chemical cross linker. The cell lysates were probed with the anti-FLAG antibody. The single asterisk and the double asterisk indicate the bands for trimer and monomer of mutant gp160, respectively. Marker: HiMark Pre-Stained High Molecular Weight Protein Standard (Invitrogen), Mock: mock transfection. [file 1742-4690-7-95-S2.TIFF]

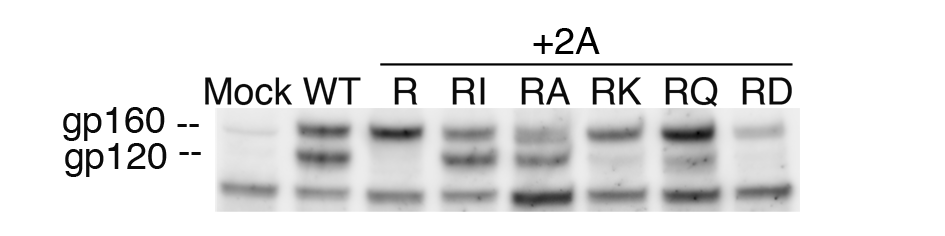

Supplement: Additional file 3 — Suplemental Figure 3A - Immunoblotting analysis of the Arg-substitution mutants in the context of 695+2A. The degree of processing of gp160 was examined by immunoblotting the cell lysates prepared from COS-7 cells transfected with respective Env expression vectors. The Arg residue in the context of 695+2A was substituted with the indicated amino acid residue by the site directed mutagenesis (columns under 2A). One letter abbreviation for an amino acid residue is used. Mock: mock transfection, WT: wild type MSD. [file 1742-4690-7-95-S3.PNG]

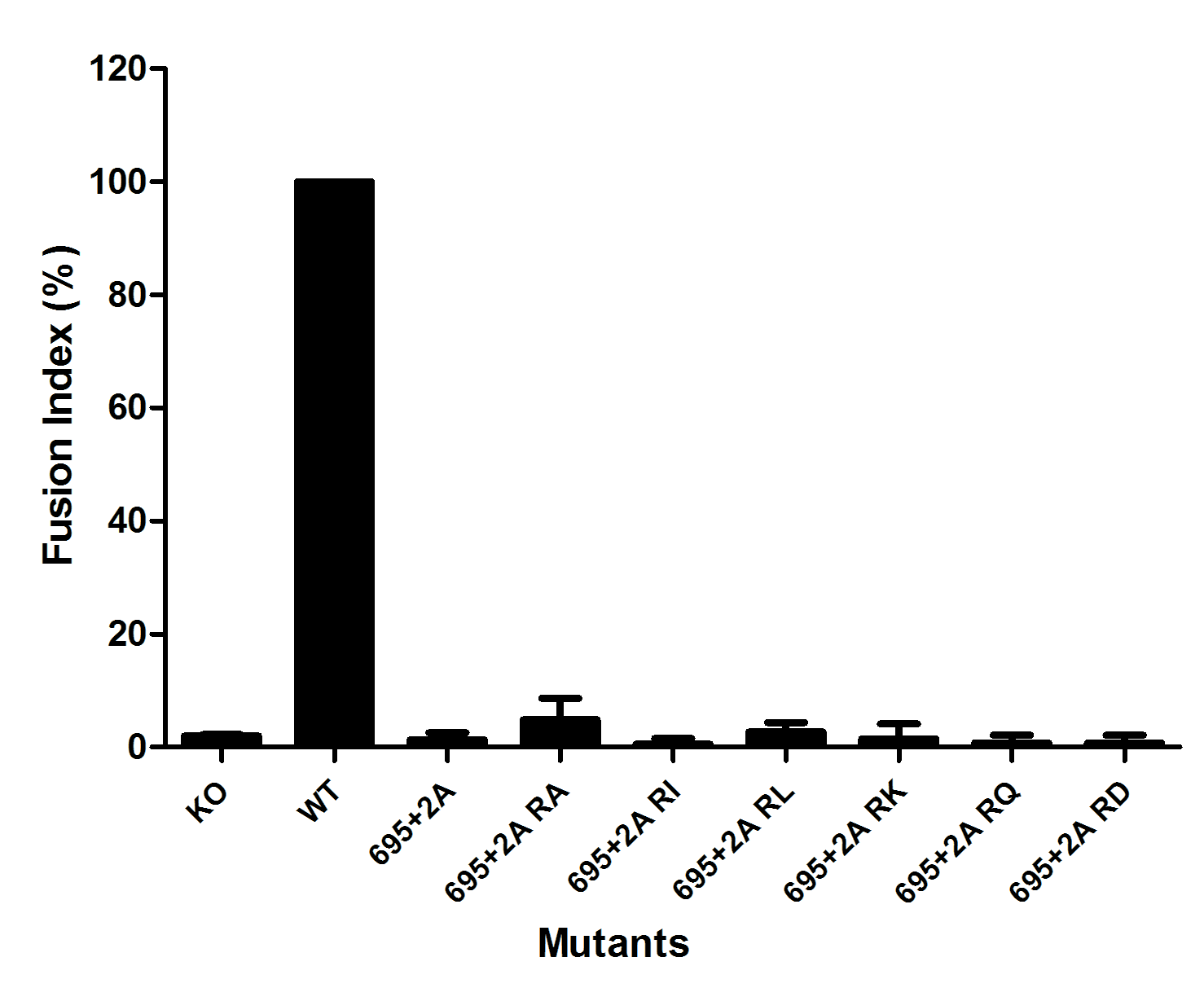

Supplement: Additional file 4 — Suplemental Figure 3B - Fusion activities of Arg-substitution mutants in the context of 695+2A. The fusion activities of the mutant shown in additional file 3A were examined by a syncytia formation assay in 293CD4 cells. Fusion activity of the WT and MSD mutants was expressed using a fusion index (fusion index = 2x + y, where x is the number of multinucleated cells [number of nuclei ≥ 5 in five visual fields] and y is the number of multinucleated cells [number of nuclei < 5 in five visual fields]) as described previously [18]. [file 1742-4690-7-95-S4.TIFF]
